# Luteolin Inhibits Fibrillary β-Amyloid_1–40_-Induced Inflammation in a Human Blood-Brain Barrier Model by Suppressing the p38 MAPK-Mediated NF-κB Signaling Pathways

**DOI:** 10.3390/molecules22030334

**Published:** 2017-02-24

**Authors:** Jun-Xia Zhang, Jian-Guo Xing, Lin-Lin Wang, Hai-Lun Jiang, Shui-Long Guo, Rui Liu

**Affiliations:** 1 Institute of Materia Medica, Chinese Academy of Medical Sciences and Peking Union Medical College, Beijing 100050, China; zhangjunxia@imm.ac.cn (J.-X.Z.); wanglinlin@imm.ac.cn (L.-L.W.); jianghailun@imm.ac.cn (H.-L.J.); 2 Key Laboratory of Uighur Medicine of Xinjiang Uygur Autonomous Region, Xinjiang Institute of Materia Medica, Urumqi 830004, China; xjguodd@163.com; 3 Department of Gastroenterology, Beijing Friendship Hospital, Capital Medical University, Beijing 100050, China; 4 National Clinical Research Center for Digestive Disease, Beijing 100050, China; 5 Beijing Key Laboratory for Precancerous Lesion of Digestive Disease, Beijing 100050, China

**Keywords:** Alzheimer’s disease, amyloid-β peptides, cyclooxygenase-2, cytokine, fibrillar amyloid-β peptides, luteolin, mitogen-activated protein kinases, nuclear factor κB, blood-brain barrier

## Abstract

Amyloid-β peptides (Aβ) exist in several forms and are known as key modulators of Alzheimer’s disease (AD). Fibrillary Aβ (fAβ) has been found to disrupt the blood-brain barrier (BBB) by triggering and promoting inflammation. In this study, luteolin, a naturally occurring flavonoid that has shown beneficial properties in the central nervous system, was evaluated as a potential agent to preserve barrier function and inhibit inflammatory responses at the BBB that was injured by fAβ_1–40_. We established an in vitro BBB model by co-culturing human brain microvascular endothelial cells (hBMECs) and human astrocytes (hAs) under fAβ_1–40_-damaged conditions and investigated the effect of luteolin by analyzing cellular toxicity, barrier function, cytokine production and inflammation-related intracellular signaling pathways. Our results demonstrated that, in cells injured by fAβ_1–40_, luteolin increased cell viability of hBMECs and hAs. The cytoprotection of the co-culture against the damage induced by fAβ_1–40_ was also increased at both the apical and basolateral sides. Luteolin protected the barrier function by preserving transendothelial electrical resistance and relieving aggravated permeability in the human BBB model after being exposed to fAβ_1–40_. Moreover, in both the apical and basolateral sides of the co-culture, luteolin reduced fAβ_1–40_-induced inflammatory mediator and cytokine production, including cyclooxygenase-2 (COX-2), tumor necrosis factor α (TNF-α), interleukin 1 β (IL-1β), interleukin 6 (IL-6), and interleukin 8 (IL-8), however it did not show sufficient effects on scavenging intracellular reactive oxygen species (ROS) in hBMECs and hAs. The mechanism of BBB protection against fAβ_1–40_-induced injury may be related to the regulation of inflammatory signal transduction, which involves inhibition of p38 mitogen-activated protein kinase (MAPK) activation, downregulation of phosphorylated inhibitory κB kinase (phosphor-IKK) levels, relief of inhibitory κB α (IκBα) degradation, blockage of nuclear factor κB (NF-κB) p65 nuclear translocation, and reduction of the release of inflammatory cytokines. Moreover, the employment of p38 MAPK and NF-κB inhibitors reversed luteolin-mediated barrier function and cytokine release. Taken together, luteolin may serve as a potential therapeutic agent for BBB protection by inhibiting inflammation following fAβ_1–40_-induced injury.

## 1. Introduction

Alzheimer’s disease (AD) is the most common form of dementia and has a high morbidity and mortality [1]. AD is characterized by progressive cognition dysfunction and neuronal cell loss and is associated with brain deposition of senile plaques and accumulation of neurofibrillary tangles [1]. Although multiple symptoms are associated with AD, accumulation of amyloid-β peptides (Aβ) is hypothesized to trigger a pathogenic cascade that eventually results in AD [2]. It is well documented that fibrillary forms of Aβ may serve as an inflammatory stimulus for neuroinflammatory responses and the underlying mechanisms have been explored in a variety of studies [3,4,5,6]. Increasing evidence suggests that components of the blood-brain barrier (BBB) can be highly responsive to inflammation caused by different forms of Aβ [7,8,9], which could either potentially contribute to events leading to subsequent neurodegeneration, or act as inflammatory mediators to potentiate the deleterious neuroinflammatory cycle. Therefore, a better understanding of the processes and mechanisms that result in Aβ-related inflammation of the BBB may lead to novel therapeutics to combat AD.

The BBB is a unique anatomical structure that is essential for maintaining homeostasis of the brain parenchymal microenvironment [10]. Brain microvascular endothelial cells (BMECs) and astrocytes (As) as the main cells of the BBB are among the key players in the brain inflammatory response that is initiated by various inflammatory events in the brain’s environment. In both rodents and humans, any Aβ form has been shown to have an effect on the BBB, which can lead to a cascade of events including alteration of the BBB permeability, oxidative stress, release of inflammatory components and disruption of the integrity [7,11,12,13,14]. Fibrillary Aβ (fAβ), a toxic Aβ peptide, has been shown to induce inflammatory effects on astrocytes, cerebral endothelial cells in Alzheimer brain as well as in a co-culture of these two cell types [4,5,15,16]. In this context, it is believed that inflammation results from Aβ toxicity, which causes the release of pro-inflammatory mediators, such as cyclooxygenase-2 (COX-2) and pro-inflammatory cytokines, such as tumor necrosis factor α (TNF-α) and interleukin 1 β (IL-1β). This may result in adverse events of the systemic and central nervous systems leading to aggravating Aβ-mediated neurodegeneration [17]. The expression of inflammation-related genes is controlled at both the transcriptional and post-transcriptional level by intracellular signaling pathways, including nuclear factor κB (NF-κB) and mitogen-activated protein kinases (MAPKs) [18,19].

**Figure 1:**
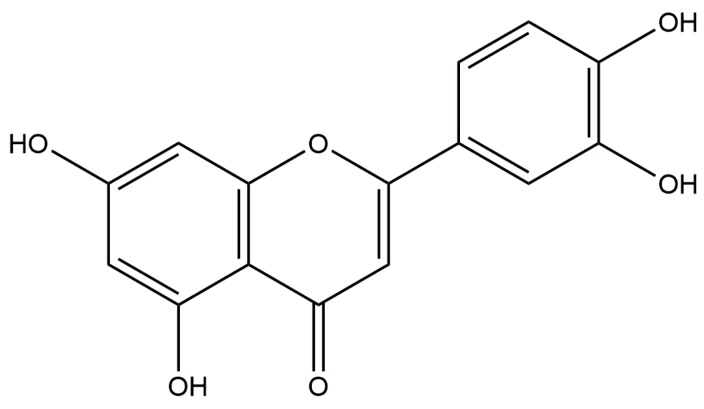
Chemical structure of luteolin.

Luteolin (3′,4′,5,7-tetrahydroxyflavone, Figure 1) is a naturally occurring flavonoid that has been shown to have anti-inflammatory, antioxidant, and neuroprotective properties. It is present in a glycosylated form in various fruits, vegetables, and medicinal herbs such as *Lonicera japonica* and *Dracocephalum moldavica*. Luteolin has been shown to inhibit the lipopolysaccharide (LPS)-induced production of TNF-α and nitric oxide (NO) in an activated macrophage-like cell line [20]. In addition, it has shown robust anti-inflammatory effects by reducing the production of LPS-induced pro-inflammatory cytokines in intestinal epithelial cells, mouse bone marrow-derived dendritic cells [21], rat fibroblasts [22], and human gingival fibroblasts [23]. Moreover, it has been demonstrated that luteolin exerted anti-amnesic effects against Aβ-induced toxicity [24,25,26]. In our previous studies, we demonstrated that the improvement in cognition and neuroprotective effects of luteolin included regulating microvascular function and protecting to BBB ultrastructure [24,27]. Although some studies suggested that, in neurodegenerative disorders, the anti-inflammatory effect of luteolin may be due to suppressing the NF-κB, MAPK, and protein kinase B (PKB) pathways in activated microglial cells [28,29], little is known about the role of luteolin in barrier protection and the possible mechanism of action during inflammatory processes at the BBB.

In the present study, we established an in vitro BBB model by co-culturing human BMECs (hBMECs) and human astrocytes (hAs) under fibrillar Aβ 1–40 (fAβ_1–40_)-induced conditions. We investigated the effect of luteolin on barrier function, oxidative stress and inflammatory responses at the BBB caused by fAβ_1–40_ and explored the potential mechanism of action in the prevention of AD progression.

## 2. Results

### 2.1. Luteolin Protects hBMECs, hAs, and Co-Culture against fAβ_1–40_-Induced Toxicity

**Figure 2:**
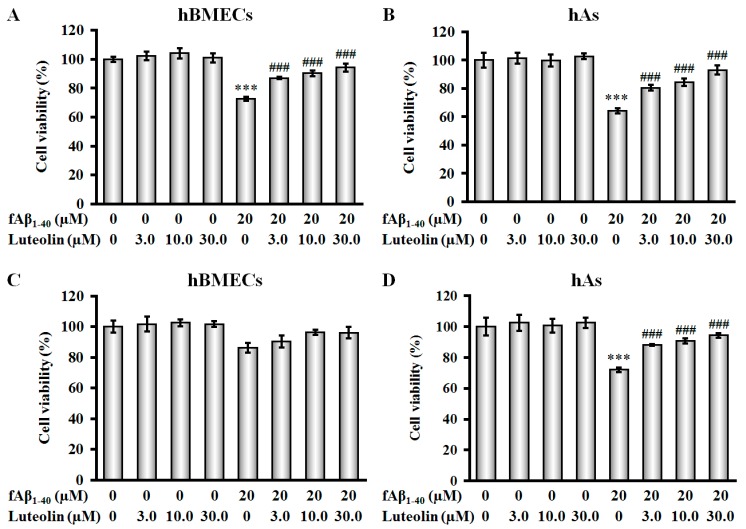
Cytoprotective effects of luteolin on human brain microvascular endothelial cells (hBMECs), human astrocytes (hAs) and co-culture against fibrillary amyloid-β peptide 1-40 (fAβ_1–40_)-induced toxicity. (**A**) Luteolin increases cell viability of hBMECs as evaluated by a 3-(4,5-dimethylthiazol-2-yl)-5-(3-carboxymethoxy-phenyl)-2-(4-sulfophenyl)-2*H*-tetrazolium (MTS) assay. *n* = 8; (**B**) Luteolin increases cell viability of hAs as evaluated by MTS assay. *n* = 8; (**C**) Luteolin has a mild effect on cell viability of hBMECs in the co-culture as evaluated by Alamar blue assay. *n* = 6; (**D**) Luteolin influences cell viability of hAs in the co-culture as evaluated by Alamar blue assay. *n* = 6. Data are expressed as the mean ± standard error of mean (SEM); *** *p* < 0.001 vs. control; ^###^ *p* < 0.001 vs. fAβ_1–40_.

Previous studies have shown that in the BBB, high concentrations of fAβ are toxic to microvascular endothelial cells and astrocytes [4,7,16]. In the present study, changes in cell viability of hBMECs and hAs were evaluated using a 3-(4,5-dimethylthiazol-2-yl)-5-(3-carboxymethoxy-phenyl)-2-(4-sulfophenyl)-2*H*-tetrazolium (MTS) assay. We found that, after exposure to 20 µM fAβ_1–40_ for 72 h, cell viability of hBMECs and hAs was reduced by 27.3% and 35.6%, respectively (Figure 2A,B, both *p* < 0.001). Administration of luteolin at doses of 3.0 µM, 10.0 µM and 30.0 µM significantly protected against fAβ_1–40_-induced cytotoxicity in both hBMECs and hAs in a concentration-dependent manner (Figure 2A,B, all *p* < 0.001). In addition, the effects of luteolin on hBMECs and hAs without fAβ_1–40_ treatment after 72 h were not significant at any of the concentrations evaluated.

To evaluate the protective effect of the BBB in the presence of luteolin, we established an in vitro co-culture model of hBMECs and hAs by applying fAβ_1–40_ to the basolateral side and luteolin to the apical side of the model. Figure 2D shows that when fAβ_1–40_ was applied to the basolateral side, there was a noticeable decrease in astrocyte viability as determined by Alamar blue assay (*p* < 0.001). Moreover, addition of luteolin at doses of 3.0 µM, 10.0 µM and 30.0 µM to the apical side of the co-culture increased cell viability compared with fAβ_1–40_-injured cells (all *p* < 0.001). A mild reduction in hBMEC viability was observed in the basolateral side of the model (Figure 2C). In addition, luteolin caused an increase in hBMEC viability; however, this effect was not significant. Luteolin did not show any significant effect on viability of hBMECs and hAs in co-culture at the tested doses after 72 h (Figure 2C,D).

### 2.2. Luteolin Improves BBB Function But Does Not Scavenge Intracellular Reactive Oxygen Species against fAβ_1–40_-Induced Toxicity

**Figure 3:**
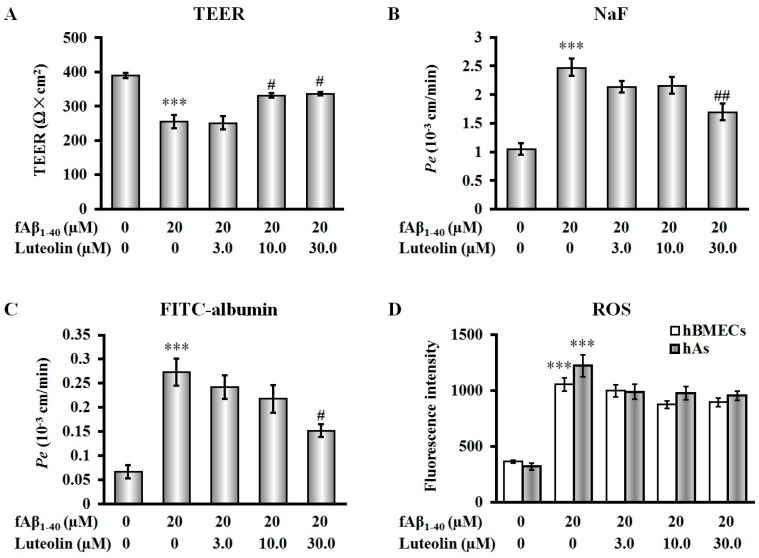
Luteolin improves blood-brain barrier (BBB) function but does not scavenge intracellular reactive oxygen species (ROS) in co-culture against fAβ_1–40_-induced toxicity. (**A**) Luteolin decreased the transendothelial electrical resistance (TEER) value at concentrations of 10.0 μmol/L and 30.0 μmol/L; (**B**) Luteolin decreased the transendothelial permeability for fluorescein sodium (NaF) by preserving the reduction in value of the endothelial permeability coefficient (*Pe*) at a concentration of 30.0 μmol/L; (**C**) Luteolin decreased fluorescein isothioyanate labeled albumin (FITC-albumin) indicated by *Pe* value at a concentration of 30 μmol/L; (**D**) Luteolin does not reduce intracellular ROS levels in hBMECs and hAs in co-culture against fAβ_1–40_-induced toxicity. Data are expressed as mean ± SEM; *n* = 4; *** *p* < 0.001 vs. control; ^#^ *p* < 0.05; ^##^ *p* < 0.01; vs. fAβ_1–40_.

To determine the effects of luteolin on barrier function injured by fAβ_1–40_, we measured transendothelial electrical resistance (TEER) and the flux of fluorescein sodium (NaF) and fluorescein isothioyanate labeled albumin (FITC-albumin), which respectively indicate the barrier integrity of the BBB and paracellular permeability. As shown in Figure 3A–C, loss of barrier integrity and increase in permeability were induced after fAβ_1–40_ was added to the basolateral side of the co-culture (all *p* < 0.001). When luteolin was applied to the apical side of the co-culture in combination with simultaneous addition of fAβ_1–40_ to the basolateral side, protective effects of barrier integrity were observed in a concentration-dependent manner in the increase of TEER at 10.0 µM and 30.0 µM (Figure 3A, both *p* < 0.05). Similarly, treatment with luteolin at a dose of 30.0 µM ameliorated BBB permeability by preserving the reduction in values of the endothelial permeability coefficient (*Pe*) of NaF and FITC-albumin (Figure 3B,C, *p* < 0.05 and *p* < 0.01, respectively).

Aβ exerts toxicity against cells of the BBB via enhanced reactive oxygen species (ROS) production and redox imbalance [4]. In this study, fAβ_1–40_ increased endogenous ROS production in hBMECs and hAs by a 2.91-fold and 3.83-fold increase, respectively (*p* < 0.001, Figure 3D). However, we found that, in the present model, luteolin could not significantly scavenge the ROS production at each concentration, which indicated that luteolin did not provide sufficient effect on ameliorating the antioxidative ability in cells of the BBB subjected to fAβ_1–40_-induced toxicity.

### 2.3. Luteolin Inhibits the Release of Inflammatory Cytokines and the Expression of COX-2 Against fAβ_1–40_-Induced Toxicity

**Figure 4:**
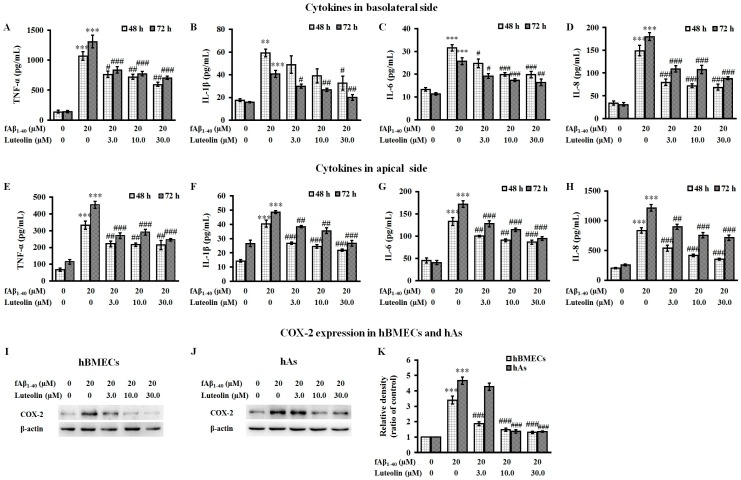
Luteolin inhibits the release of inflammatory cytokines and the expression of cyclooxygenase-2 (COX-2) against fAβ_1–40_-induced toxicity. Luteolin decreased the levels of tumor necrosis factor α (TNF-α) (**A**); interleukin 1 β (IL-1β) (**B**); interleukin 6 (IL-6) (**C**) and interleukin 8 (IL-8) (**D**) in the basolateral supernatant and the levels of TNF-α (**E**); IL-1β (**F**); IL-6 (**G**); and IL-8 (**H**) in the apical supernatant collected at 48 h and 72 h time points following treatment with fAβ_1–40_. Data are expressed as the mean ± SEM; *n* = 4; ** *p* < 0.01; *** *p* < 0.001 vs. control; ^#^ *p* < 0.05; ^##^ *p* < 0.01; ^###^ *p* < 0.001 vs. fAβ_1–40_. Luteolin also down-regulated the expression of COX-2 in cell extracts of hBMECs (**I**) and hAs (**J**) of the co-culture after exposure to fAβ_1–40_ for 72 h. Relative density values of COX-2 were quantified to intensity levels of β-actin (**K**). Data are expressed as the mean ± SEM, *n* = 4; *** *p* < 0.001 vs. control; ^###^ *p* < 0.001 vs. fAβ_1–40_.

To determine whether fAβ_1–40_ treatment has inflammatory effects in a co-culture model of human BBB, we investigated alterations in cytokine secretion and COX-2 expression at both the apical and basolateral sides of the co-culture following treatment with fAβ_1–40_. We found that in the apical and basolateral supernatant, BBB cells secreted very low levels of TNF-α, IL-1β, IL-6 and IL-8. Administration of fAβ_1–40_ to the basolateral side of the co-culture caused a significant increase in the production of pro-inflammatory cytokines, such as TNF-α, IL-1β, IL-6, and IL-8, in the supernatant collected at 48 h and 72 h of both the apical and basolateral sides of the co-culture (Figure 4A–H, *p* < 0.01 or *p* < 0.001).

When luteolin was added for 72 h to the apical side of the co-culture, the release of pro-inflammatory cytokines stimulated by fAβ_1–40_ was significantly inhibited over the 48 to 72 h time period (Figure 4A–H, *p* < 0.05–*p* < 0.001). The presence of luteolin caused a reduction of fAβ_1–40_-stimulated pro-inflammatory cytokines in the apical side that was similar as the reduction seen in the basolateral side. After 72 h of fAβ_1–40_ stimulation, the expression of COX-2 in cell extracts of both hBMECs and hAs was significantly increased (Figure 4I–K, both *p* < 0.001). In response to fAβ_1–40_-induced up-regulation of COX-2 in the BBB, luteolin was found to reduce the expression of COX-2 by hBMECs in a concentration-dependent manner from 3.0 µM to 30.0 µM (all *p* < 0.001). In addition, the expression of COX-2 by hAs was also inhibited when administered at doses of 10.0 and 30.0 µM (both *p* < 0.001).

### 2.4. Luteolin Inhibits NF-κB and MAPK Signal Pathways against fAβ_1–40_-Induced Toxicity

**Figure 5:**
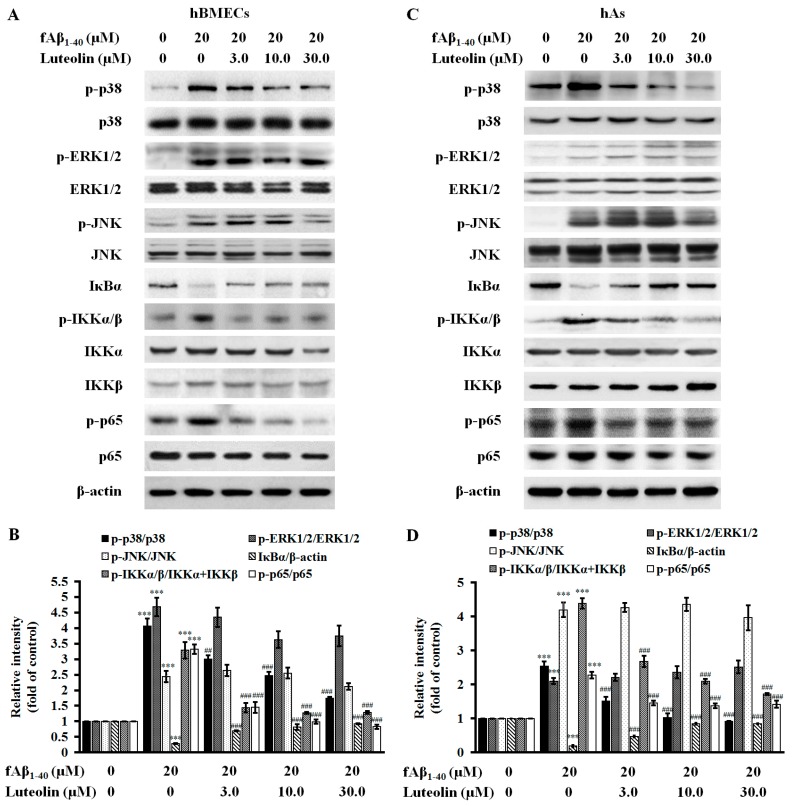
Luteolin suppresses mitogen-activated protein kinases (MAPKs) and nuclear factor κB (NF-κB) signaling pathways against fAβ_1–40_-induced toxicity. Representative immunoblots illustrate the expression of phosphorylated p38 (phosphor-p38), p38, phosphorylated extracellular signal-regulated kinase 1/2 (ERK1/2) (phosphor-ERK1/2), ERK1/2, phosphorylated c-Jun N-terminal kinase (phosphor-JNK), JNK, phosphorylated inhibitory κB kinase α/β (phosphor-IKKα/β), IKKα, IKKβ, inhibitory κB α (IκBα), phosphor-p65, and p65 in hBMECs (**A**) and hAs (**C**) extracts of the co-culture after exposure to fAβ_1–40_ for 72 h. Quantitative analysis of the above-mentioned proteins by hBMECs (**B**) and hAs (**D**) was demonstrated. Data are expressed as the mean ± SEM; *n* = 3; *** *p* < 0.001 vs. control; ^##^ *p* < 0.01; ^###^ *p* < 0.001 vs. fAβ_1–40_.

MAPK and NF-κB signaling pathways have been shown to be involved in the pathological effects of Aβ [30,31]. To determine if luteolin anti-inflammatory effects were mediated through inhibition of MAPK and NF-κB signaling pathways in the BBB, western blot analysis was performed. In the MAPK pathway, increased ratios of phosphorylated versus non-phosphorylated forms of p38 MAPK, extracellular signal-regulated kinase 1/2 (ERK1/2) and c-Jun N-terminal kinase (JNK) were observed in the hBMECs and hAs after fAβ_1–40_ administration to the co-culture (Figure 5A–D, all *p* < 0.001). In co-cultured hBMECs and hAs, luteolin inhibited phosphorylation of p38 MAPK at doses of 3.0 µM, 10.0 µM and 30.0 µM (*p* < 0.01–0.001), but did not affect phosphorylation levels of JNK and ERK1/2 that were induced by fAβ_1–40_ in hBMECs and hAs. For NF-κB-related signaling proteins, fAβ_1–40_ treatment resulted in upregulation of phosphorylation levels of inhibitory κB kinase α /β (IKKα/β) and nuclear distribution of p65 subunit in hBMECs and hAs. Levels of inhibitory κB α (IκBα) in both hBMECs and hAs of the co-culture were reduced (Figure 5A–D, all *p* < 0.001). Luteolin treatment showed a beneficial effect on inhibiting IKKα/β phosphorylation, IκBα degradation and subsequent p65 phosphorylation at defined concentrations in hBMECs and hAs (all *p* < 0.001), suggesting that luteolin is effective in suppressing the activation of NF-κB signaling in response to fAβ_1–40_.

### 2.5. Involvement of the p38 MAPK/NF-κB Pathway in the Protective Effect of Luteolin on fAβ_1–40_-Induced Barrier Function and Inflammation

**Figure 6:**
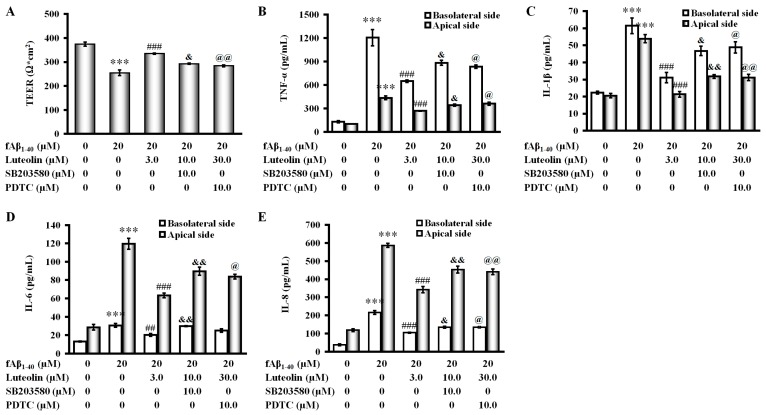
Effects of inhibition of p38 MAPK and NF-κB on the effect of luteolin on fAβ_1–40_-induced barrier dysfunction and inflammation. To block p38 MAPK and NF-κB pathways, co-cultures were treated with p38 MAPK inhibitor SB203580 (4-(4-fluorophenyl)-2-(4-methylsulfinylphenyl)-5-(4-pyridyl)-1*H*-imidazole, 10.0 μM) or NF-κB inhibitor PDTC (pyrrolidine dithiocarbamate, 20.0 μM) for 30 min prior to treatment with luteolin. Abolished effects of p38 MAPK and NF-κB inhibition of the effect of luteolin on TEER value (**A**); and production of TNF-α (**B**); IL-1β (**C**); IL-6 (**D**); and IL-8 (**E**) were seen by treatment with SB203580 or PDTC. Data are expressed as the mean ± SEM; *n* = 4; *** *p* < 0.001 vs. control; ^##^ *p* < 0.01; ^###^ *p* < 0.001 vs. fAβ_1–40_; ^&^ *p* < 0.05; ^&&^ *p* < 0.01 vs. luteolin combined with fAβ_1–40_; ^@^ *p* < 0.05; ^@@^ *p* < 0.01 vs. luteolin combined with fAβ_1–40_.

To further examine the mechanisms involved in regulating fAβ_1–40_-induced inflammation in the treatment of luteolin, we added the pharmacological inhibitors of p38 MAPK and NF-κB to the co-culture when exposed to fAβ_1–40_, and investigated the participation of the p38 MAPK/NF-κB pathway in BBB function and inflammatory cytokine production. Although luteolin treatment at 30 µM increased the value of TEER and inhibited cytokine release, such as TNF-α, IL-1β, IL-6, and IL-8, in both sides in the co-culture, after subjection to fAβ_1–40_ for 72 h (Figure 6, all *p* < 0.001), these effects were significantly changed by the addition of the pharmacological p38 MAPK inhibitor, SB203580 (4-(4-fluorophenyl)-2-(4-methylsulfinylphenyl)-5-(4-pyridyl)-1*H*-imidazole; Beyotime Institute of Biotechnology, Shanghai, China) (Figure 6, *p* < 0.05—*p* < 0.01), and the NF-κB inhibitor, pyrrolidine dithiocarbamate (PDTC; Beyotime Institute of Biotechnology) (Figure 6, *p* < 0.05–*p* < 0.01).

These findings indicate that inhibition of p38 MAPK by luteolin treatment may inhibit inflammatory responses induced by fAβ_1–40_. Therefore, the p38 MAPK/NF-κB pathway might be involved in the protective effects of luteolin on the BBB.

## 3. Discussion

The goal of this study was to investigate the effect of luteolin on fAβ_1–40_-induced changes in an in vitro BBB model by analyzing cell toxicity, barrier function, ROS, and cytokine production and inflammation-related intracellular signaling pathways. The results showed that luteolin increased cell viability of hBMECs and hAs that were injured by fAβ_1–40_ treatment. In the human BBB model, luteolin also protected barrier function by preserving the TEER value and by reducing aggravated permeability after exposure to fAβ_1–40_. Luteolin did not show significant effects on scavenging intracellular ROS in hBMECs and hAs of the co-culture. However, luteolin reduced fAβ_1–40_-induced inflammatory mediator and cytokine production in both the apical and basolateral sides of the co-culture. The mechanism of this opposite effect by fAβ_1–40_ may be related to the regulation of inflammatory signaling transduction at the BBB, involving suppression of p38 MAPK activation, downregulation of phosphor-IKK levels, relief of IκBα degradation, inhibition of NF-κB p65 nuclear translocation and reduction of the release of inflammatory cytokines. Furthermore, employment of p38 MAPK and NF-κB inhibitors reversed luteolin-mediated barrier function and cytokine release. Therefore, luteolin may be a potential therapeutic agent for BBB protection by inhibiting inflammation against fAβ_1–40_-induced injury.

It is known that the BBB is formed by the continuous brain microvascular endothelium, which depends on the underlying basement membrane, pericytes that ensheathe the endothelial wall, and astrocytes in the surrounding tissue space that extend their cell processes towards the endothelium [32]. Together, endothelial cells, pericytes, and astrocytes are all required for the maintenance of normal physiology of the neurovasculature and maintenance of BBB integrity [32,33]. It has been generally known that signaling and interaction between pericytes and endothelial cells is critical for BBB structure and function [34]. In addition, pericytes have been shown to control key neurovascular functions that are necessary for proper neuronal function. Therefore, dysfunction of pericytes results in a progressive age-dependent vascular-mediated neurodegeneration [34]. Importantly, the present study is part of our ongoing screening program to evaluate the neuroprotective potential of natural compounds on endothelial cells and astrocytes to the response of the BBB [8,24,27]. Based on our previous findings, we used the static BBB model with human BMECs and astrocytes cultured in Transwell plates that would permit analysis of the independent contributions of luteolin on hBMECs and hAs to the response of the BBB to inflammation induced by Aβ.

In AD, a number of different forms of Aβ peptides are present. Monomeric, oligomeric and fibrillary Aβ toxicity to endothelial cells of cerebrovascular origin has been claimed [13,15,16]. Previous reports have shown that, in both animal models and in cell culture models, fAβ caused cell death in BBB endothelial cells [4,35]. In the present study, we illustrated that fAβ_1–40_ at a higher concentration (20 μM) induced significant cell toxicity on the BBB by reducing viability not only in the hBMEC or hAs as a monolayer, but also in the endothelial cells of the co-culture model, which is in line with other studies in which was shown that fAβ induced death and dysfunction of BBB-related cells [16]. Abnormally, the reduction of viability was not apparent in hBMECs under a co-culture system. The major cause of this phenomenon is attributable to the application fAβ to the basolateral side of this system; therefore, the passive crossings of brain and systemic Aβ via a leaky BBB induced by structure failure or permeability probably fall behind the Aβ transport via BBB receptors or transporters. Furthermore, several studies in human brain endothelial cell lines have demonstrated that treatment of cells in combination of monomeric and oligomeric Aβ_1–42_ resulted in toxicity, however viability remained unaffected [12]. In terms of these supports, we deduce that accompanied by a mild viability reduction of endothelial cells in the co-culture, functional and molecular changes from endothelial toxicity must be induced following basolateral application of fAβ.

In response to fAβ_1–40_-induced toxicity, the effective administration conditions for luteolin were determined using both control and fAβ_1–40_-injured hBMECs and hAs. Luteolin administration was evaluated at doses of 3.0 µM, 10.0 µM and 30.0 µM, and was found to significantly increase the viability of both cell types injured with fAβ_1–40_. Moreover, no significant differences were found among luteolin treatment in control cells, indicating that, under basal conditions, luteolin has no toxic effects within the evaluated concentrations. In addition, an integrative co-culture of both the vascular and glial compartment was employed to assess the protective effect of luteolin on pathological changes in the BBB. At the concentrations tested, luteolin treatment protected hBMECs and hAs in a similar fashion, which is consistent with the effects seen on hBMECs or hAs monolayers against fAβ_1–40_-induced toxicity. Therefore, these data confirmed that luteolin protects the cells that form the human BBB model from fAβ_1–40_-induced toxicity.

Under physiological conditions, the BBB controls the entry of plasma-derived Aβ into the central nervous system (CNS) by the receptor for advanced glycation end products (RAGE)-mediated re-entry transport [36,37,38]. In addition, brain-derived Aβ in the plasma are cleared by low density lipoprotein receptor related protein 1 (LRP-1)-mediated scavenging signal transduction [39]. Given that in plasma, increased levels of free Aβ were identified in AD patients as well as in AD mouse models [40,41], the accumulation of Aβ peptides is believed to be a causative event in cerebrovascular alterations [42]. In the co-culture model of the human BBB established in this study, fAβ_1–40_ was added to the basolateral side of the co-culture model. This stemmed from our consideration that an initial increase of Aβ in brain parenchyma can trigger a vicious cycle of BBB damage and induce an efflux of abluminal Aβ to blood vessels in AD pathology. Our data indicated that treatment with a concentration of 20 μM fAβ_1–40_ for 72 h induced changes on the BBB, impairing barrier integrity and increasing the paracellular permeability. These findings are in line with other studies showing Aβ-induced cell death and dysfunction of BBB-associated components [16,43].

Luteolin possesses multiple beneficial properties throughout the entire CNS. To mimic the route from capillary to brain parenchyma, in our study, luteolin was applied to the apical side of the co-culture, and simulates the capability of passing through the BBB [44]. Considering these results, luteolin not only protected against fAβ_1–40_-induced cell death but also inhibited BBB dysfunction. Collectively, this would contribute towards the direct protection of the BBB from the fAβ_1–40_-induced compromised barrier. TEER is an important indicator of barrier tightness of inter-endothelial tight junctions. Reduction of the TEER value was found to be remarkably attenuated by treatment with high concentrations of luteolin, indicating that the preservation of barrier integrity may contribute to brain protection against fAβ_1–40_-induced damage. In addition, at the highest concentration, luteolin treatment decreased apical-to-basolateral diffusion of NaF and FITC-albumin. Given that luteolin partly alleviated the paracellular permeability after exposure to fAβ_1–40_, we assumed that a suitable loss of “tightness” may help therapeutic agents cross the BBB. Previous studies have reported the preservation of TEER value and transendothelial permeability of luteolin on BBB function when vascular endothelial cells were subjected to other forms of Aβ [25,28]. In our previous studies, we showed that luteolin preserved cerebral microvascular endothelial cells in the presence of Aβ_25–35_, involving the relief of TEER reduction, the increase of characteristic enzymatic activity and the regulation of secretion of inflammatory mediators [27]. Moreover, luteolin was reported to maintain microvascular function in Aβ_25–35_ intra-cerebroventricular-injected mice, in response to the decrease of regional cerebral blood flow values and the disruption of microvascular ultrastructures [24]. In our ongoing evaluation of Aβ, we provide evidence that luteolin is effective in protecting the human BBB by maintaining barrier integrity, and by regulating BBB permeability.

In AD, cerebral amyloid angiopathy (CAA) is often complicated and is caused by the deposition of Aβ along the walls of the cerebral vasculature, which includes arteries, arterioles, veins and less often capillaries [45,46]. This leads to disruption of blood vessels, disturbed cerebral blood flow and inflammatory infiltration at the BBB [47]. At the BBB, astrocytes that have a distinctive anatomical location by covering the endothelial cells of the BBB form a network of fine lamellae closely opposed to the outer surface of the endothelium [35], and can act as buffers in the brain, protecting neurons from harmful chemicals, ROS, COX-2 and cytokines [48]. As the BBB is comprised of specialized brain endothelial cells that contribute to CNS homeostasis by restricting entry of circulating leukocytes and blood-borne molecules into the CNS, BBB constitutive endothelial cells are liable to respond to inflammation associated with brain-specific injuries and disorders, such as AD, ischemia and hypoxia, or Parkinson’s disease [49]. In our study, fAβ_1–40_ was found to be the causative factor from the basolateral side. Pro-inflammatory cytokines and mediators, such as TNF-α, IL-1β, IL-6, IL-8, and COX-2, were increased in response to fAβ_1–40_ induction, suggesting that the presence of fAβ_1–40_ at the basal membrane (brain-side) may have an inflammatory effect in the absence of endothelial cell death or loss of endothelial barrier integrity. Furthermore, the overproduction of inflammatory cytokines and the up-regulation of COX-2 expression from the apical surface of the endothelial cells was similar to those of primary cerebral endothelial cell lines in response to various forms of Aβ [50,51], indicating that cytokine release and mediator expression are a direct effect of fAβ_1–40_ induction on endothelial cells rather than a merely downstream effect of its action on astrocytes. The clinical observations of this inflammation are likely to result in immune cell infiltration that will result in neuroinflammation in the brain and lead to greater damage. Clinical results have shown that in the vessel walls of AD patients, an increased number of monocytes/macrophages is present [52], which can be partly explained by the increased levels of cytokines and mediators from the endothelial cells in the co-culture. Moreover, microvascular endothelial cells are a rich source of inflammatory factors involving cytokines and chemokines in response to a wide variety of stimuli [53]. This is especially true in the AD brain, where on the surface of endothelial cells high levels of inflammatory mediators, such as inducible nitric oxide synthase and monocyte chemoattractant protein-1 are expressed [54,55], indicating an auto-amplified inflammatory molecular cascade at the BBB.

Luteolin has been proposed to be a potential therapeutic agent for the treatment of a variety of inflammatory diseases [56]. The beneficial benefits of luteolin in the CNS include the decrease of inflammation and axonal damage by preventing monocyte migration across the BBB [57]. Inhibition of the neuroinflammatory effect of luteolin was found to reduce the secretion of several pro-inflammatory enzymes and pro-inflammatory cytokines by activated microglia [28]. Subsequent studies have shown that luteolin can abolish AD-like pathological features by reducing levels of glial-derived inflammatory cytokines [58,59]. In our co-culture model, luteolin protected against fAβ_1–40_-induced cytokine release and COX-2 expression both from hAs and hBMECs and inhibited BBB compromise; taken together, this would contribute towards the protection of the BBB from fAβ_1–40_-induced barrier compromise. Regarding the effects on reducing fAβ_1–40_-stimulated apical secretion of pro-inflammatory cytokines in hBMECs, luteolin would inhibit the negative effects of cytokines and mediators on barrier integrity and immune cell infiltration as described above. Moreover, we deduce that the decrease of cytokine secretion suggests that luteolin may be able to protect endothelial cells from inflammatory insults by down-regulating the cells’ ability to initiate an inflammatory response. Luteolin also showed a cytoprotective effect on astrocytes, which was seen when this compound was applied on either the apical side of the co-culture or on the single-cultured monolayer. Due to the lipophilicity, luteolin can cross the BBB [58], and possibly have a direct protective effect on the astrocytes on the brain-side. Here, luteolin showed an anti-inflammatory effect on the astrocytes through reductions in basolateral cytokine levels and COX-2 expression. As it maintains the integrity of the BBB in the above experiments, luteolin may down-regulate the levels of cytokines and mediators from astrocytes and have a protective effect against pro-inflammatory insults induced by fAβ_1–40_.

Numerous studies have revealed that in AD brains, the preferential accumulation of Aβ in endothelial cells, reduced cerebral blood flow, transmigration of leukocytes, and secretion of multiple inflammatory factors due to injury to the BBB collectively contribute to BBB dysfunction [47]. Moreover, the induction of cytokines and increase in inflammatory signaling are early events caused by the overproduction of ROS that is triggered by various forms of Aβ [60,61]. One of the key regulators of the inflammatory processes are members of the MAPK family, involving ERK, JNK and p38 MAPK pathways. As a downstream signaling molecule of MAPK family members, NF-κB activation is regulated by MAPKs through IκB kinase activation, which induces IκB degradation [62]. In addition, NF-κB is activated by the presence of fAβ and ROS, and can upregulate pro-oxidant genes, increase cytokine release and other inflammatory and immune signaling pathways [63]. The cellular signaling events mediating Aβ-induced upregulation of inflammatory mediators has been correlated in multiple CNS cell lines using NF-κB signaling proteins, such as human endothelial cells [64], glia [65] and neurons [66]. In these resting cells, NF-κB is sequestered in the cytoplasm in an inactive form by the inhibitory IκB proteins. In response to proinflammatory mediators, an inhibitory molecule, IκBα, is phosphorylated and the NF-κB p65 component translocates to the nucleus, binds to the promoter of target genes and activates transcription of several pro-inflammatory genes [67]. Thus, suppressing NF-κB and MAPK signaling pathways directly or indirectly are proactive to anti-inflammation.

Previous studies have shown that luteolin regulates different targets through inhibition of MAPK family members in response to various stimuli [68,69]. In our study, we found that phosphorylation of p38 MAPK was downregulated by luteolin treatment. In response to fAβ_1–40_ induction, luteolin treatment did not result in a phosphorylated decrease of ERK1/2 and JNK in the BBB. p38 MAPK and ERK are the most extensively investigated members of the MAPK family mediating intracellular signaling cascades that are directly involved in NF-κB activation. In SK-N-SH neuroblastoma cells and human bronchial muscle cells, expression of inflammatory mediators induced by IL-1β alone or in combination with other cytokines was reduced through p38 MAPK inhibition [70,71]. In neuronal cells, pharmacologic inhibitors of both p38 MAPK and ERK1/2 decreased DNA binding as well as transcriptional activity of NF-κB and abolished subsequent COX-2 expression induced by Aβ [66]. Increasing evidence indicated that blocking NF-κB activation and cytokine expression can be inactivated by a dominant-negative mutation of p38 MAPK [66]. Similarly, in the p38 MAPK signaling blockade assay to ascertain luteolin-mediated protection on the BBB via the p38 MAPK pathway, we found that SB203580 significantly blocked luteolin-induced protective effects on the barrier function accompanied by the overproduction of proinflammatory cytokines in fAβ_1–40_-stimulated co-culture. Therefore, we assume that inhibiting p38 MAPK activity at the BBB may contribute to luteolin’s inhibitory effect on cytokine production in response to fAβ_1–40_.

In this study, our data showed that luteolin inhibited NF-κB signaling pathways, including upregulation of phosphor-IKK and downregulation of IκBα in both cell types of the BBB co-culture. These findings are consistent with the inhibitory effects of luteolin on p38 MAPK. In addition, luteolin decreased the expression of phosphor-p65, suggesting that luteolin can block the translocation of p65 from the cytosol to the nucleus. Increasing evidence indicated that blocking NF-κB activation and cytokine expression can be inactivated by dominant-negative mutations of p38 MAPK [66], therefore, we assumed that NF-κB along with MAPKs may participate in amplifying the loop of inflammatory responses after BBB cells were subjected to fAβ_1–40_-induction. This provided experimental proof for using an NF-κB inhibitor. We further found that NF-κB inhibition by the inhibitor PDTC reversed the effects of luteolin on fAβ_1–40_-induced BBB dysfunction and cytokine release. That is, p38 MAPK inhibitor SB203580 or NF-κB PDTC achieved almost the same results. Therefore, we may conclude that luteolin exhibits its protective effects against fAβ_1–40_-induced injury by p38 MAPK-mediated NF-κB signaling pathways under fAβ_1–40_ condition in the human BBB co-culture.

**Figure 7:**
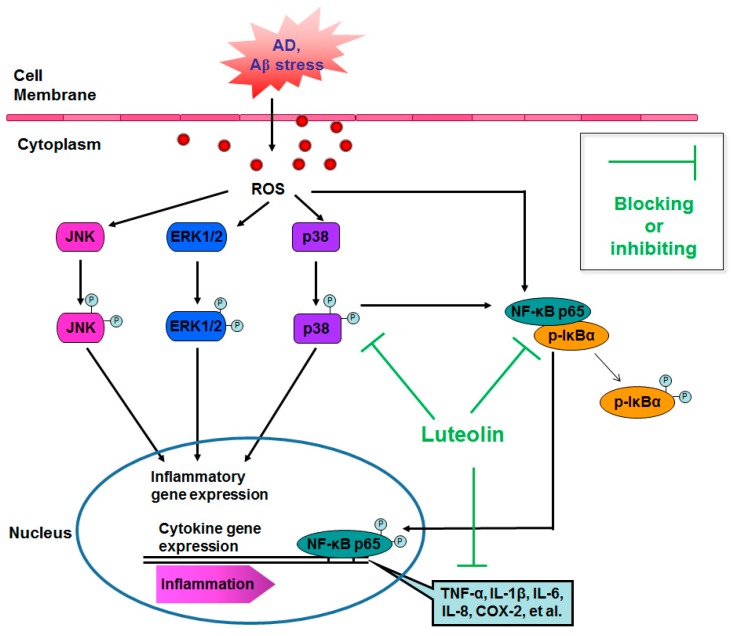
Possible mechanisms by which luteolin inhibits fibrillary β-amyloid_1–40_-induced inflammation in a BBB model. AD: Alzheimer’s disease; Aβ stress: amyloid-β peptide induced cellular stress; NF-κB p65: nuclear factor κB p65 subunit; p-IκBα: phosphorylated inhibitory κB α.

A redox homeostasis balance plays a key pathological role in the process of early inflammatory cellular signaling of AD. Aβ-mediated ROS generation is closely associated with the activation of MAPK and NF-κB signaling cascades [72]. Our previous studies have revealed that luteolin is beneficial in protecting microvascular endothelial cells and in maintaining the integrity of the BBB via scavenging ROS and repairing redox imbalance in conditions that are rich in Aβ [24,27]. However, in this study, we found that luteolin merely produced a slight decrease in the effectiveness of ROS generation in endothelial and astrocytic cultures in combination with fAβ. In general, the protective capacity of flavonoids against different insults has been attributed to their antioxidant potency [73,74]. Nonetheless, cytoprotection of flavonoids was no more defined to be correlated with the antioxidation potency [75]. Many flavonoids show an important pharmacological effect on modulating the activities of protein kinases, lipid kinases and enzymes of mitochondrial respiratory chain independent of their antioxidant capacity [76,77,78]. Although anti-oxidation is not involved in the critical mechanisms that prevent fAβ-mediated toxicity at the BBB, the results obtained in this study demonstrate that p38 MAPK and NF-κB are key inhibitory signaling components that are regulated by the treatment of luteolin. Our future studies will focus on unraveling the mechanisms of luteolin acting on MAPK and NF-κB signaling cascades (Figure 7).

## 4. Materials and Methods

### 4.1. Reagents

Luteolin (98% HPLC purity) was provided by Prof. Jian-Guo Xing from the Xinjiang Institute of Materia Medica of China (Urumqi, China). Luteolin was isolated from total flavonoids extracted from the aerial part of *Dracoephalum moldavica* L. (Patent No. CN 200710203385.1; specimen ID 20100708). Cell culture plastics were purchased from Corning (Corning Co., Corning, NY, USA). All other reagents were purchased from Sigma Chemical Company (St. Louis, MO, USA) unless stated otherwise.

### 4.2. Cell Culture and Treatment

hBMECs and hAs were purchased from ScienCell Research Laboratories (ScienCell Research Laboratories, Carlsbad, CA, USA). Cells were initially expanded in 75 cm^2^ flasks that were pre-coated with fibronectin (3 μg/cm^2^) and poly-d-lysine (3 μg/cm^2^), respectively. The hBMECs were cultured in endothelial cell complete medium supplemented with 10% endothelial cell growth supplement (ECGS; ScienCell Research Laboratories, Carlsbad, CA, USA) and 10% fetal bovine serum (FBS; Gibco/Invitrogen, Grand Island, NY, USA). hAs were expanded in Dulbecco’s modified essential medium (DMEM/F12; Gibco/Invitrogen) supplemented with 2 mM glutamine and 10% FBS (Gibco/Invitrogen) according to the manufacturer’s guidelines. Both cell lines were maintained at 37 °C in a humidified atmosphere with 5% CO_2_. Experiments were conducted at cell passages 4–6. All treatments were performed at a confluency of 80%–90%.

Synthetic Aβ_1–40_ was purchased from Sangon Biotech Company (Shanghai, China) and dissolved in sterile water to prepare a stock solution of 0.1 mM to foster the fibrillization state, as previously reported [16,79]. Luteolin was initially dissolved in 100 mM of DMSO and then diluted in culture medium at final concentrations of 3.0 μM, 10.0 μM, and 30.0 μM. At the start of fAβ_1–40_-initiated injury, different concentrations of luteolin were added and incubated for 72 h. hBMECs and hAs were randomly divided into the following groups: (1) control group; (2) control group treated with 3 μM luteolin; (3) control group treated with 10.0 μM luteolin; (4) control group treated with 30 μM luteolin for 72 h; (5) fAβ_1–40_ group treated with 20 μM fAβ_1–40_ for 72 h; (6) fAβ_1–40_ group treated with 3 μM; (7) 10 μM; and (8) 30.0 μM luteolin for 72 h.

### 4.3. Co-Culture of hBMECs and hAs

hAs were plated on the bottom of 0.1% gelatin-coated 24-well Transwell inserts and incubated for 6 h. After hAs had adhered, inserts were placed upright into 24-well plates, containing 900 μL of DMEM/F12 medium supplemented with 10% FBS. hBMECs were plated on top of the insert in endothelial cell complete medium supplemented with FBS and endothelial cell growth supplement. Next, 20 μM fAβ_1–40_ was added to the basolateral side of inserts (astrocyte side) and 22–24 h after plating the hBMECs, 3.0 μM, 10.0 μM and 30.0 μM of luteolin was added to the well insert (endothelial side). Similarly, for investigating the roles of the p38 MAPK and NF-κB pathways in fAβ_1–40_-induced injury rescued by luteolin, the specific p38 MAPK and NF-κB inhibitors, SB203580 (10.0 μM) or PDTC (20.0 μM), were added for 30 min prior to treatment with luteolin and fAβ stimulation.

### 4.4. MTS Cell Viability Assay

Cell survival rates were assessed using the MTS assay (Promega, Madison, WI, USA). MTS assays were performed in accordance with the manufacturer’s guidelines. A SpectraMax Plus microplate reader was used for detection (Molecular Devices Corp., Sunnyvale, CA, USA).

### 4.5. Alamar Blue Cell Viability Assay

Co-cultures of hBMECs and hAs were established as described above. After fAβ_1–40_ and luteolin treatments, 10% Alamar Blue solution (Life Technologies, Grand Island, NY, USA) was added to both the co-culture insert and 24-well dish, respectively. Four hours later, 100 μL of medium was transferred to a clean 96-well plate and fluorescence was measured using a plate reader at excitation/emission wavelengths of 540/590 nm. Cell free medium was included as a negative control.

### 4.6. TEER Measurement

The value of TEER indicates the integrity and viability of tissue culture bilayers. TEER was measured using an Electronic Volt-Ohmmeter resistance meter (World Precision Instruments, Sarasota, FL, USA). The extracellular matrix-treated Transwell inserts (Corning Co.) were placed in a 12-well plate containing culture medium and background resistance was determined. Background resistance was subtracted from filters containing cells. TEER values were expressed as Ω × cm^2^.

### 4.7. Transendotheial Permeability Measurements

Permeability to NaF (molecular weight: 376 Da) and FITC-albumin (molecular weight: 67 kDa) was determined as described previously [80]. Briefly, co-cultures were established in 12 mm diameter Transwell inserts and treated as described above. At completion of the assay, inserts were transferred to 12-well plates containing Ringer solution (118 mM NaCl, 4.8 mM KCl, 2.5 mM CaCl_2_, 1.2 mM MgSO_4_, 5.5 mM glucose, and 20 mM Hepes, pH 7.4). In the apical chamber, culture medium was replaced by 500 μL Ringer solution containing 10 μg/mL NaF and 165 μg/mL FITC-albumin. The inserts were transferred to separate wells at 20 min, 40 min and 60 min. Basolateral chamber solutions were collected and fluorescence was measured at excitation/emission wavelengths of 488 nm/535 nm and 525 nm/440 nm using a microplate reader (Molecular Devices Corp, Sunnyvale, CA, USA). In addition, the flux across cell-free inserts was measured. *Pe* value was calculated as previously described [81].

### 4.8. Enzyme-Linked Immunosorbent Assay for Proinflammatory Cytokines

At the time points of fAβ_1–40_, luteolin and/or inhibitor treatment, culture medium in both the apical and basolateral chambers was collected and centrifuged for 5 min at 4 °C and 1000 rpm. Supernatants were collected for enzyme-linked immunosorbent assay (ELISA) for cytokines. Human ELISA kits (eBioscience, San Diego, CA, USA) were used for the detection of TNF-α, IL-1β, IL-6, and IL-8 and were performed according to the manufacturer’s instructions.

### 4.9. Western Blot Analysis

Following treatments, hBMECs and hAs were collected from the inserts and prepared for western blot analysis. For lysis of the cells, RIPA buffer (Cell Signaling Technology, Danvers, MA, USA) was used, containing protease inhibitors, phosphatase inhibitors and phenylmethanesulfonyl fluoride (PMSF). After determination of the protein concentration, proteins were separated on a 10% sodium dodecyl sulfate-polyacrylamide gel, and transferred to polyvinylidene fluoride (PVDF) membranes using a semidry transfer system (Bio-Rad, Hercules, CA, USA). Membranes were blocked with 5% non-fat milk in Tris-buffered saline, containing 0.05% Tween-20 (TBST) for 1 h at room temperature and incubated overnight with the following primary antibodies: rabbit anti-ERK1/2 (1:1000, Cell Signaling), rabbit anti-p-ERK1/2 (1:1000, Cell Signaling), rabbit anti-JNK (1:1000, Cell Signaling), rabbit anti-p-JNK (1:1000, Cell Signaling), rabbit anti-p38 MAPK (1:1000, Cell Signaling), rabbit anti-p-p38 MAPK (1:1000, Cell Signaling), rabbit anti-IKKα (1:1000, Cell Signaling), rabbit anti-IKKβ (polyclonal antibody, 1:1000, Cell Signaling), rabbit anti-p-IKKα/β (1:1000, Cell Signaling), rabbit anti-IκBα (1:1000, Cell Signaling), rabbit anti-NFκB-p65 (1:1000, Cell Signaling), rabbit anti-p-NFκB-p65 (1:1000, Cell Signaling), rabbit anti-COX-2 (1:1000, Cell Signaling), and mouse anti-β-actin (1:2000, Cell Signaling). Membranes were washed with TBST prior to incubation with horseradish peroxidase (HRP)-conjugated secondary antibody (1:1000, ZSGB-Bio, Beijing, China) at room temperature. After subsequent washes in TBST, final detection was performed using an enhanced chemiluminescence (ECL) detection kit (GE Healthcare, Piscataway, NJ, USA). The density of each band was quantified using an image densitometer. As a loading control, the membranes were blotted against β-actin (1:2000, Cell Signaling). The expression COX-2 and IκBα were normalized by β-actin intensity and phosphorylation levels were normalized by the non-phosphorylated form of MAPKs and NF-κB.

### 4.10. Intracellular ROS Detection

ROS levels were detected by 2′,7′-dichlorofluorescin diacetate (DCFDA; Abcam, Cambridge, MA, USA), which is oxidized by intracellular ROS to 2′,7′-dichlorofluorescein (DCF), a highly fluorescent compound. Briefly, 10 μL of cell homogenates of each treatment group was mixed with 90 μL of phosphate buffer (pH 7.4) in black 96-well plates and treated with 50 μM DCFDA per well. Fluorescence intensity was measured by a microplate reader (Molecular Devices Corp., Sunnyvale, CA, USA) using an excitation wavelength at 485 nm and emission wavelength at 535 nm.

### 4.11. Statistical Analysis

Data are represented as the mean ± standard error of mean (SEM). *p* values of less than 0.05 were considered statistically significant. Statistical analyses were performed using SPSS software (Version 16.0, SPSS, Inc., Chicago, IL, USA). Comparisons were performed by one-way ANOVA followed by a Tukey’s multiple comparison post-hoc test.

## 5. Conclusions

In conclusion, the present study demonstrates that fAβ_1–40_-induced cytotoxicity and barrier dysfunction at the BBB can be rescued by luteolin treatment. The protective effects on the BBB against fAβ_1–40_ exhibited by luteolin are mediated through anti-inflammatory effects. The mechanisms involved may include inhibition of p38 MAPK activation, reduction of phosphor-IKK activation, suppression of IκBα degradation, blockage of NF-κB p65 nuclear translocation, reduction of proinflammatory cytokine release, and COX-2 expression. Furthermore, the employment of p38 MAPK and NF-κB inhibitors reversed luteolin-mediated barrier function and cytokine release. Taken together, the suppression of p38 MAPK-mediated NF-κB pathway may play a significant role in endothelial cell and astrocyte protection of luteolin at the BBB. Given its effects on barrier protection and crossing of the BBB, luteolin may serve as a potential therapeutic agent in the prevention and/or treatment of AD.
